# The m.13051G>A mitochondrial DNA mutation results in variable neurology and activated mitophagy

**DOI:** 10.1212/WNL.0000000000002688

**Published:** 2016-05-17

**Authors:** Eszter Dombi, Alan Diot, Karl Morten, Janet Carver, Tiffany Lodge, Carl Fratter, Yi Shiau Ng, Chunyan Liao, Rebecca Muir, Emma L. Blakely, Iain Hargreaves, Mazhor Al-Dosary, Gopa Sarkar, Simon J. Hickman, Susan M. Downes, Sandeep Jayawant, Patrick Yu-Wai-Man, Robert W. Taylor, Joanna Poulton

**Affiliations:** From the University of Oxford (E.D., A.D., K.M., J.C., T.L., C.L., R.M., S.M.D., J.P.); Churchill Hospital (C.F.), Oxford; Newcastle University (Y.S.N., E.L.B., M.A.-D., P.Y.-W.-M., R.W.T.), Newcastle upon Tyne; National Hospital for Neurology and Neurosurgery (I.H.), UCLH, Queen Square, London; Stoke Mandeville Hospital (G.S.), Aylesbury; Royal Hallamshire Hospital (S.J.H.), Sheffield; John Radcliffe Hospital (S.J.), Oxford; Royal Victoria Infirmary (P.Y.-W.-M.), Newcastle upon Tyne; and Moorfields Eye Hospital and UCL Institute of Ophthalmology (P.Y.-W.-M.), London, UK.

Maternally inherited mitochondrial DNA (mtDNA) mutations cause symptoms of Leber hereditary optic neuropathy (LHON) in ∼1 in 30,000 individuals. Most of the affected individuals lack respiratory chain defects^1^ and there is no proven prophylactic treatment.

We identified 2 families (figure 1A) and 1 singleton case (appendix e-1 on the *Neurology*® Web site at Neurology.org) harboring the m.13051G>A pathogenic mtDNA mutation.^2^ This mutation was homoplasmic (figure e-1) but no respiratory chain defect was apparent in skeletal muscle (figure e-2, table e-1). Three children were severely affected by lactic acidosis: 2 with Leigh syndrome (patients 1 and 2; figure 1B) and 1 with a Leigh-like phenotype (patient 5). Previous authors have shown that mtDNA and mitochondrial mass are increased in individuals harboring LHON mutations.^3^ They suggested that an upregulation of mitochondrial biogenesis is protective, as the highest mitochondrial content was found in symptom-free carriers.^3^ We believe this increase in biogenesis reflects heightened mitochondrial turnover and therefore investigated mitophagy, a cellular mechanism whereby redundant or dysfunctional mitochondria are recycled.

**Figure 1:**
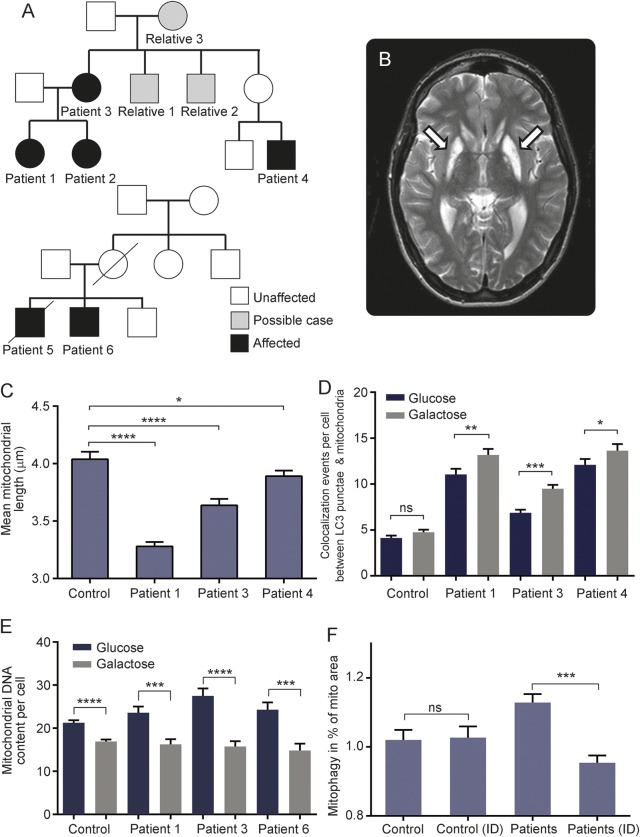
Clinical data and evidence of activated mitophagy in patients with m.13051G>A mutation (A) Family trees of patients with the m.13051G>A mutation. All maternally related individuals harbored the m.13051G>A mitochondrial DNA (mtDNA) mutation, but only those shaded black are clinically affected. Three children were severely affected with lactic acidosis: 2 with Leigh syndrome (patients 1 and 2) and one with a Leigh-like phenotype (patient 5). For further clinical details, see appendix e-1. (B) T2-weighted axial brain MRI scan head of patient 2. Arrows show established, bilateral, and symmetrical areas of hyperintensity in the lentiform nuclei, consistent with Leigh disease. (C) Mean mitochondrial length (measured using the IN Cell1000 Analyzer, GE Life Sciences, Piscataway, NJ) was significantly decreased in the patient cell lines compared with controls. (D) Number of mitophagic events was significantly increased in all patient cell lines carrying the m.13051G>A mtDNA mutation. An even more marked degree of mitophagy activation was observed when the cells were stressed under conditions of energetic deprivation induced by culture in galactose media; this was not significant in the control. Cells were grown in glucose or galactose media (represented by blue or gray bars, respectively) and measured using in Cell1000 Analyzer. (E) MtDNA content was mildly increased in the patient cell lines compared with controls (n = 3) under glucose media conditions (NS). There was a significant reduction in mtDNA content when the cells were grown in galactose media. (F) We investigated the effect of idebenone (a synthetic analogue of coenzyme Q10) on mitophagy in both control and patient cells (n = 4) by adding 100 μL of idebenone (ID, 1 μM final concentration) to the growth media. Idebenone led to a significant reduction in the levels of mitophagy in the m.13051G>A mutant cell lines, suggestive of a beneficial effect on overall mitochondrial function (*p ≤ 0.05, **p ≤ 0.01, ***p ≤ 0.001, ****p ≤ 0.0001; one-way analysis of variance with multiple comparison, error bars are SEM).

## Methods.

We used IN Cell1000, a previously developed high-throughput imaging method for quantifying mitophagy and mtDNA^4^ in cultured fibroblasts from patients compared with cultures derived from karyotypically normal disease controls aged 0–20 years and healthy volunteers aged 21–80 years. Cells were immunostained for the autophagy marker LC3 and the mitochondrial protein TOM20. Mitophagy was assessed as colocalization of LC3 punctae with TOM20-positive mitochondria.

## Results.

We found that the m.13051G>A mutation occurred on the background of 3 different clades (table e-2), suggesting that this has arisen on multiple occasions. We established that fibroblasts from all patients have fragmented mitochondrial network (figure 1C) along with elevated levels of mitophagy (figure 1D) when compared to controls. Mitochondrial volume was also increased, as was reactive oxygen species (ROS) production, accompanied by an increase in the mitochondrial antioxidant manganese superoxide dismutase (figures e-3 and e-4).

Changing cell culture substrates from glucose to glucose-free (galactose) media forced cells to use oxidative phosphorylation and further increased levels of mitophagy (figure 1D). An increase in LC3 punctae colocalized with mitochondria does not distinguish between slowed degradation of autophagosomes and increased flux unless it is validated, for example by mtDNA content. The increase in mitophagy was accompanied by a drop in mtDNA content (figure 1E), suggesting increased turnover. Treatment of m.13051G>A patient cells with idebenone attenuated the increase in mitophagy (figure 1F).

## Discussion.

Combining our data with 2 previously published cases,^2^ the m.13051G>A mtDNA mutation appears to have arisen independently several times, cosegregating with clinical features of either classical LHON or a complicated early-onset Leigh-like neurodegenerative phenotype. This specific mtDNA variant was not detected in 990 control mtDNA sequences. Taken together, our genetic and functional in vitro assays firmly establish a pathogenic role for the m.13051G>A mutation in causing mitochondrial disease.

Interestingly, patient-derived fibroblasts had a fragmented mitochondrial network pointing towards an imbalance between fusion and fission. In keeping with this observation, mitochondrial mass was increased in the mutant cell lines, which is not surprising given that activation of mitochondrial biogenesis is a well-reported compensatory cellular mechanism. Furthermore, there was a significant increase in the levels of ROS production at baseline (figure e-3). Mitophagy was robustly increased in all the fibroblast cultures tested that carried the m.13051G>A mutation (figure 1D). Previously published data showed increased mitophagy in patients with complex I deficient mtDNA disease.^5^ We also have additional data supporting the same effect in fibroblasts from other classical mitochondrial optic neuropathies caused by the m.11778A>G (n = 2) and m.3460G>A (n = 1) mtDNA mutations and from a patient who is a compound heterozygote for pathogenic ACAD9 mutations (figure e-5). In the absence of an overt mitochondrial respiratory chain defect, documenting increased mitophagy in fibroblasts could be a useful functional assay that would further support the pathogenic nature of a specific mtDNA variant.

The energetic stress induced by forcing the cells to use oxidative phosphorylation leads to an increase in mitophagy and a decrease in mtDNA content. This could be explained by an increase in ROS production that overcomes the antioxidative defenses resulting in mitochondrial damage and increased mitophagy. Idebenone attenuates this mitophagy and seems to improve cell viability (not shown), most likely by ameliorating respiratory chain dysfunction and limiting the production of ROS. Patients with LHON may benefit from treatment with idebenone.^6^

We have shown that mitophagy is increased in cells from patients with Leigh/LHON phenotypes secondary to the m.13051G>A mtDNA mutation. Furthermore, idebenone attenuates the increased mitophagy. Drugs that modulate mitophagy are therefore potentially useful treatments for mitochondrial and other neurodegenerative disorders.^7^

## Supplementary Material

Data Supplement
