# Supplementary material for: The m.13051G>A mitochondrial DNA mutation results in variable neurology and activated mitophagy
Source: Neurology. 2016 May 17;86(20):1921–3. doi: 10.1212/WNL.0000000000002688 (PMC4873683; doi:10.1212/WNL.0000000000002688)
Supplement: Data Supplement [file supp_86_20_1921__index.html]

Data Supplement 

# The m.13051G>A mitochondrial DNA mutation results in variable neurology and activated mitophagy

## Data Supplement

Seven figures, two tables, and one appendices; one Microsoft PowerPoint file and one Microsoft Word file.

**Neurology® data supplements are not copyedited before publication. Published editorials and translations have been copyedited.  
 © 2016 American Academy of Neurology.  
  
 Files in this Data Supplement:**

- Figures e-1 to e-7, Tables e-1 to e-2 - Microsoft PowerPoint file
- Appendix e-1 - Microsoft Word file
