# Supplementary material for: The m.13051G>A mitochondrial DNA mutation results in variable neurology and activated mitophagy
Source: Neurology. 2016 May 17;86(20):1921–3. doi: 10.1212/WNL.0000000000002688 (PMC4873683; doi:10.1212/WNL.0000000000002688)
Supplement: Data Supplement [file supp_WNL.0000000000002688_Supplementary_Figure.ppt]

## Slide 1
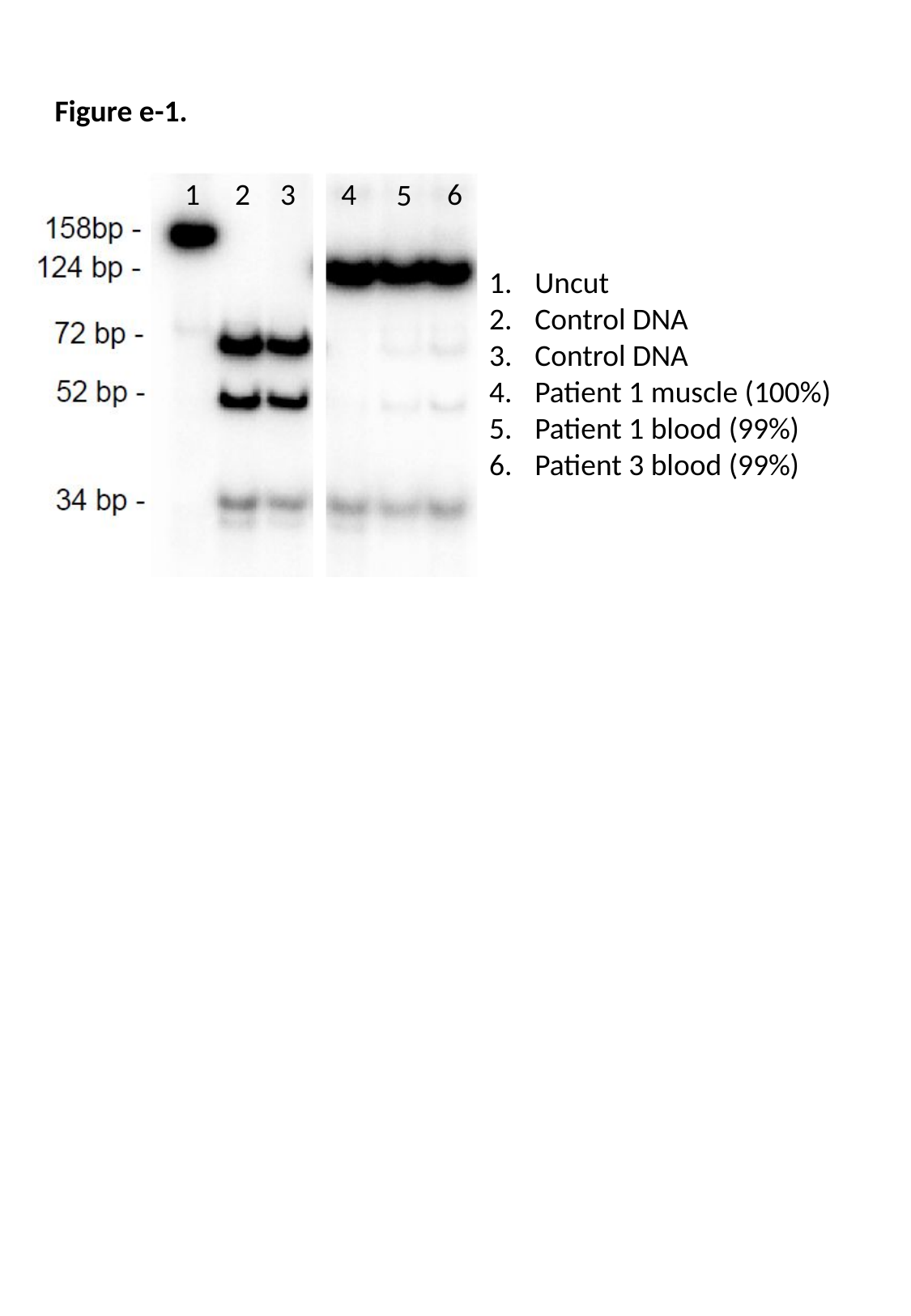

Figure e-1.
1
2
3
4
6
5
Uncut
Control DNA
Control DNA
Patient 1 muscle (100%)
Patient 1 blood (99%)
Patient 3 blood (99%)

## Slide 2
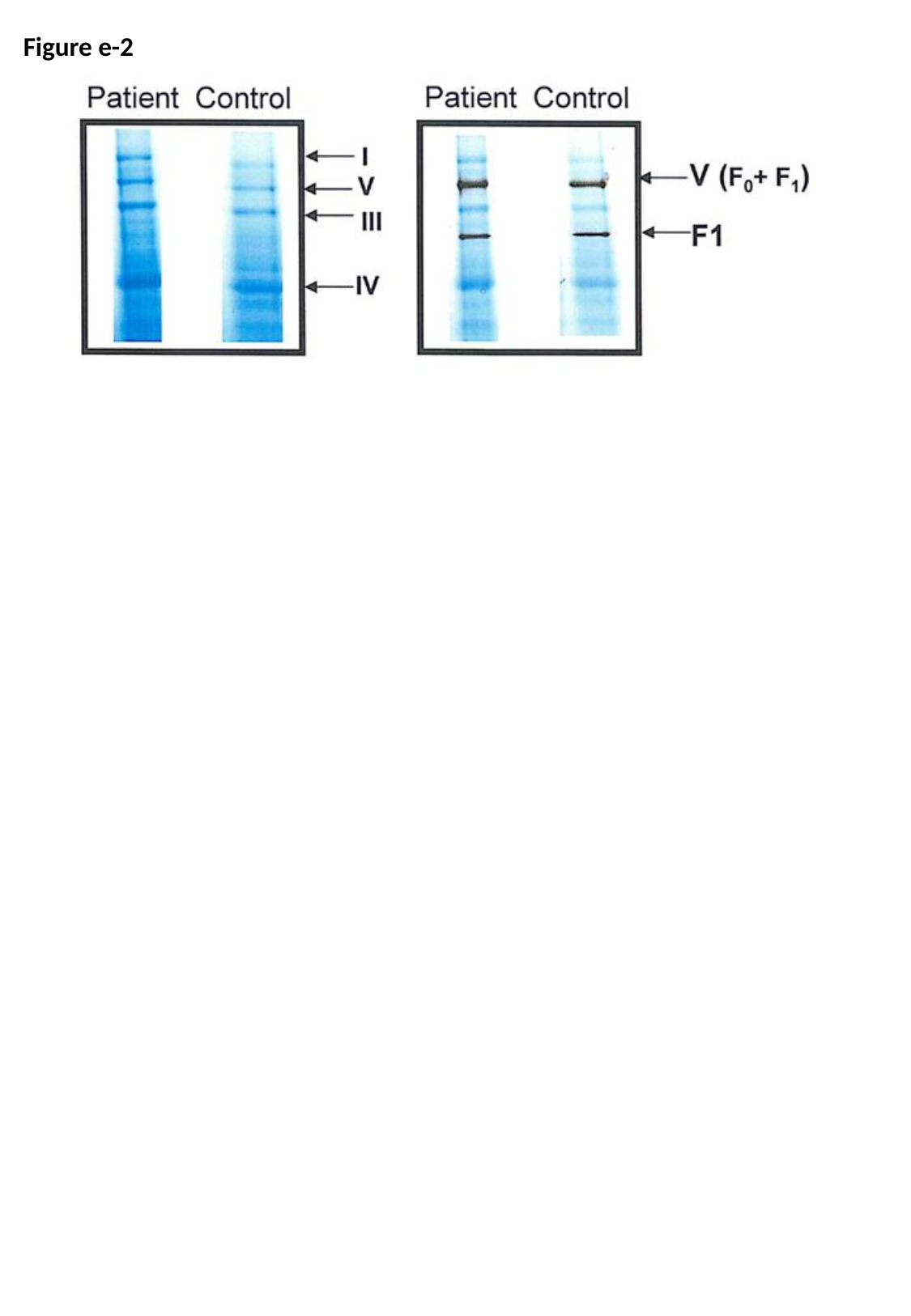

Figure e-2

## Slide 3
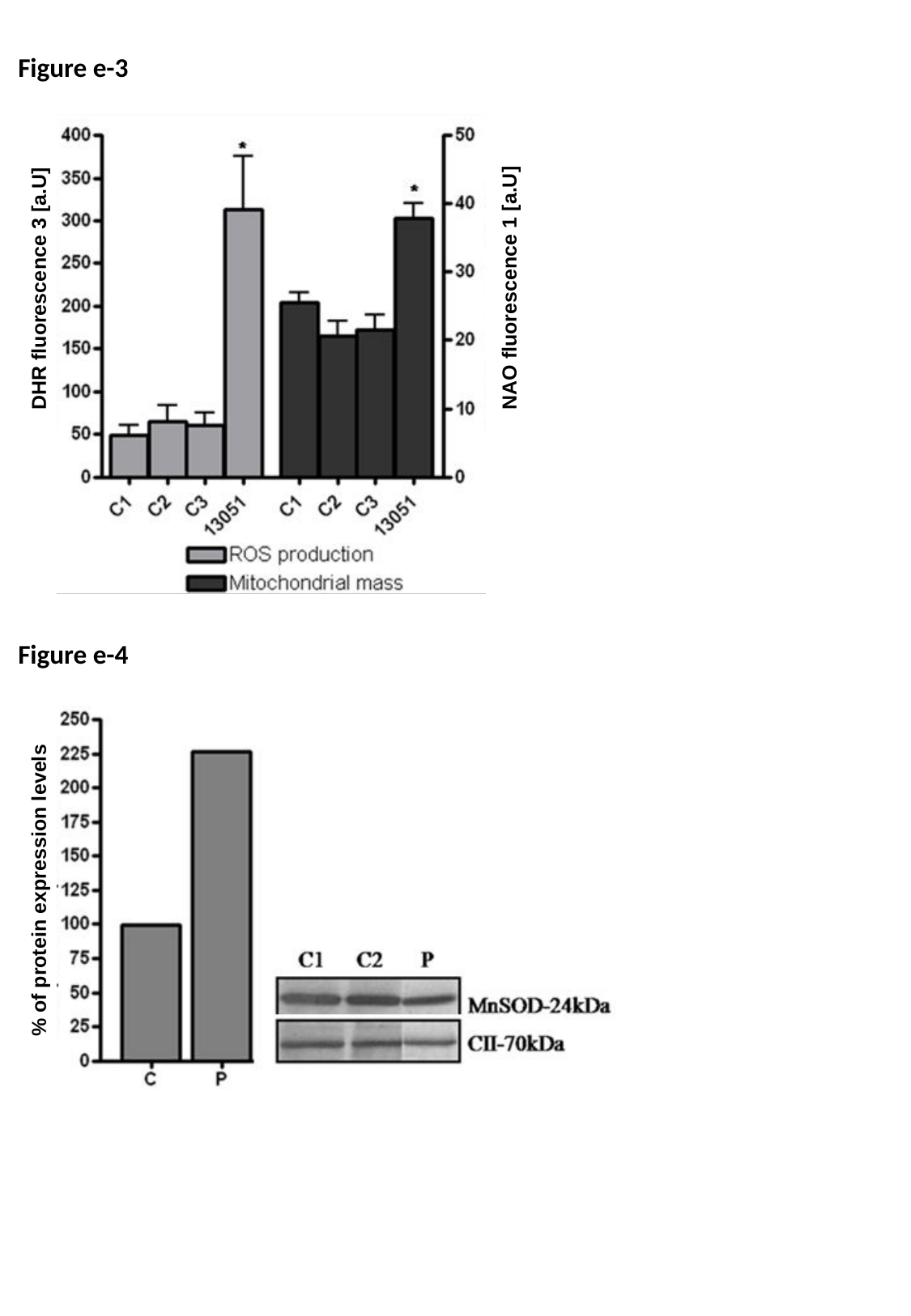

Figure e-3
DHR fluorescence 3 [a.U]
NAO fluorescence 1 [a.U]
 Figure e-4
% of protein expression levels

## Slide 4
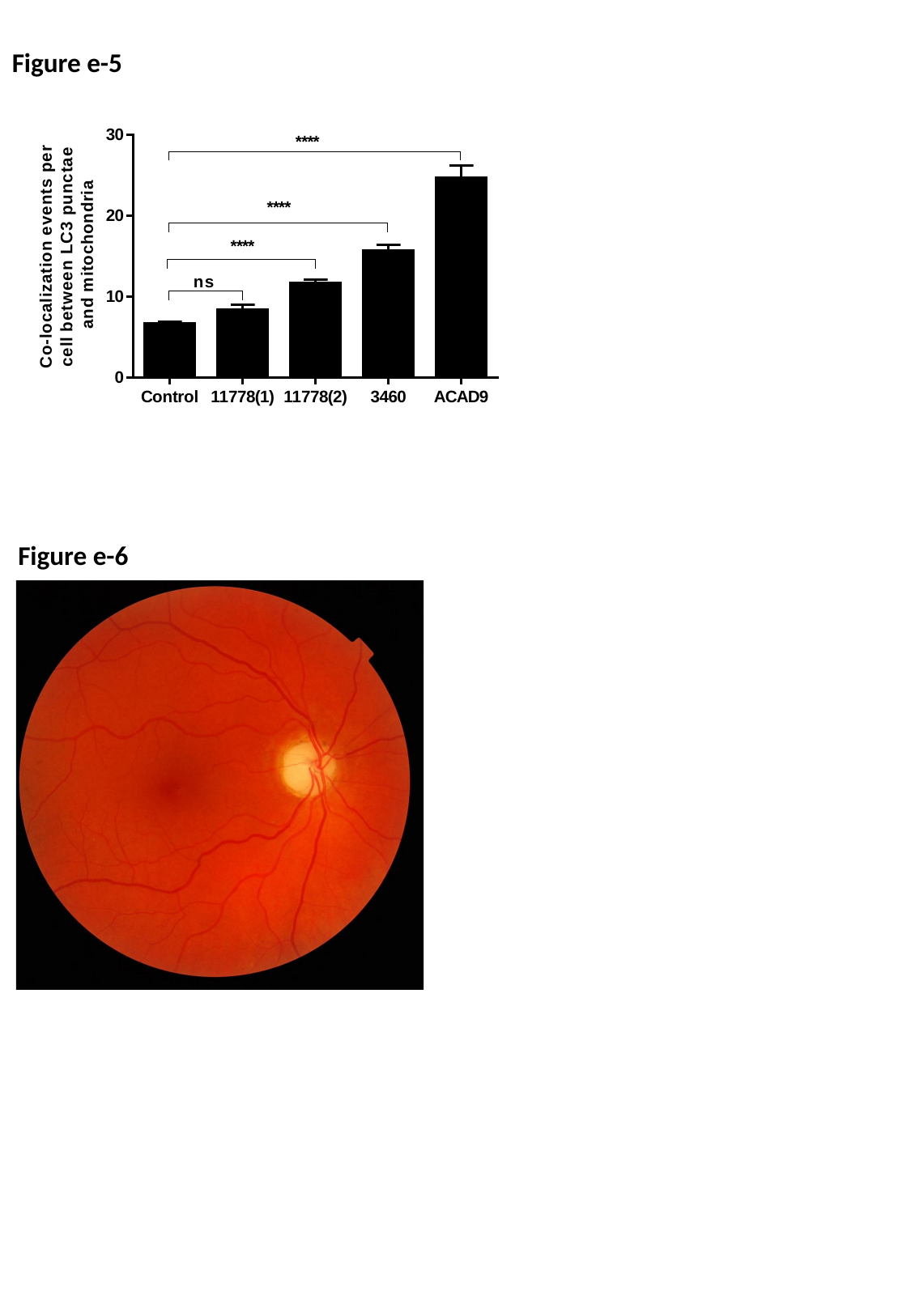

Figure e-5
 Figure e-6

## Slide 5
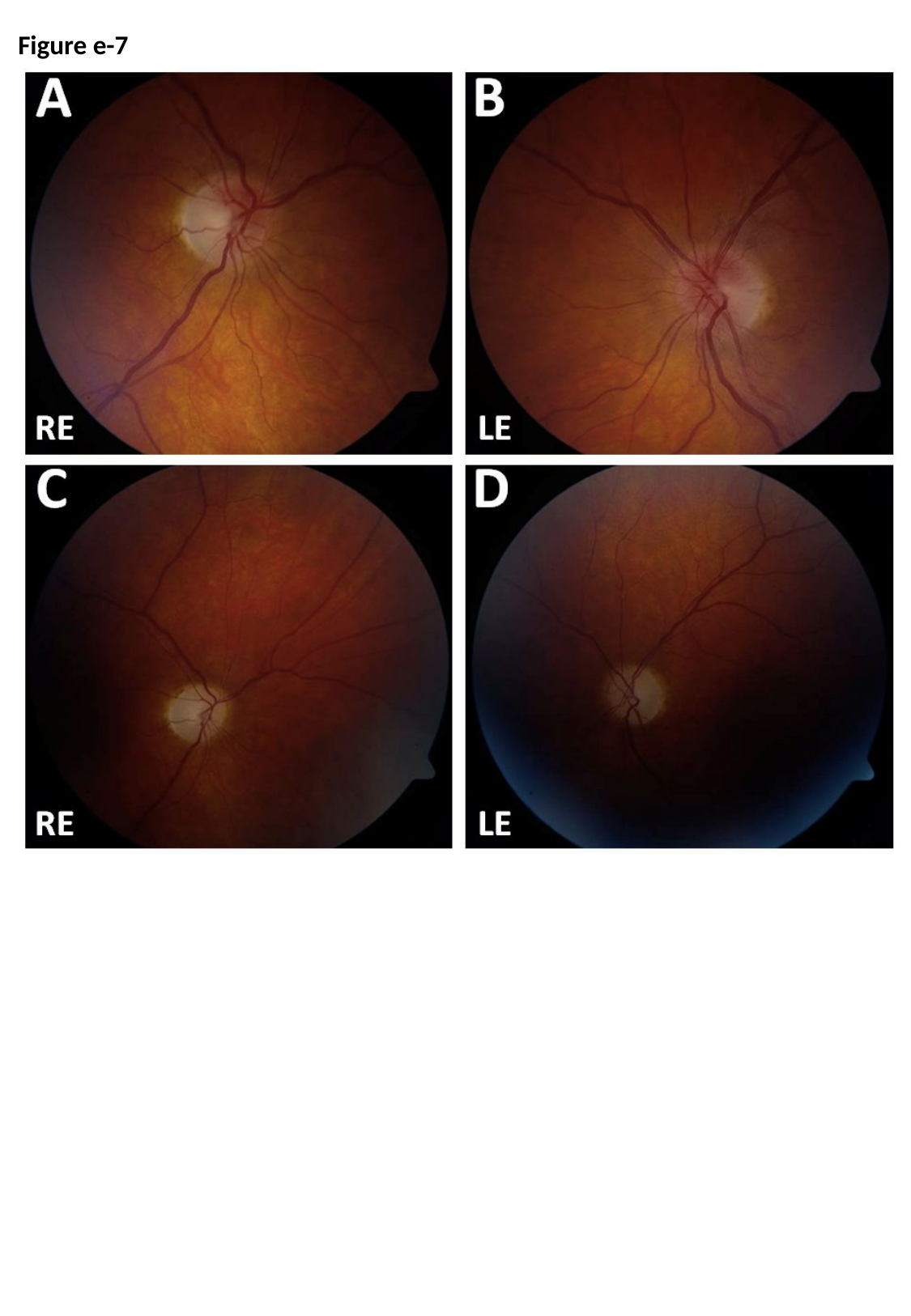

Figure e-7

## Slide 6
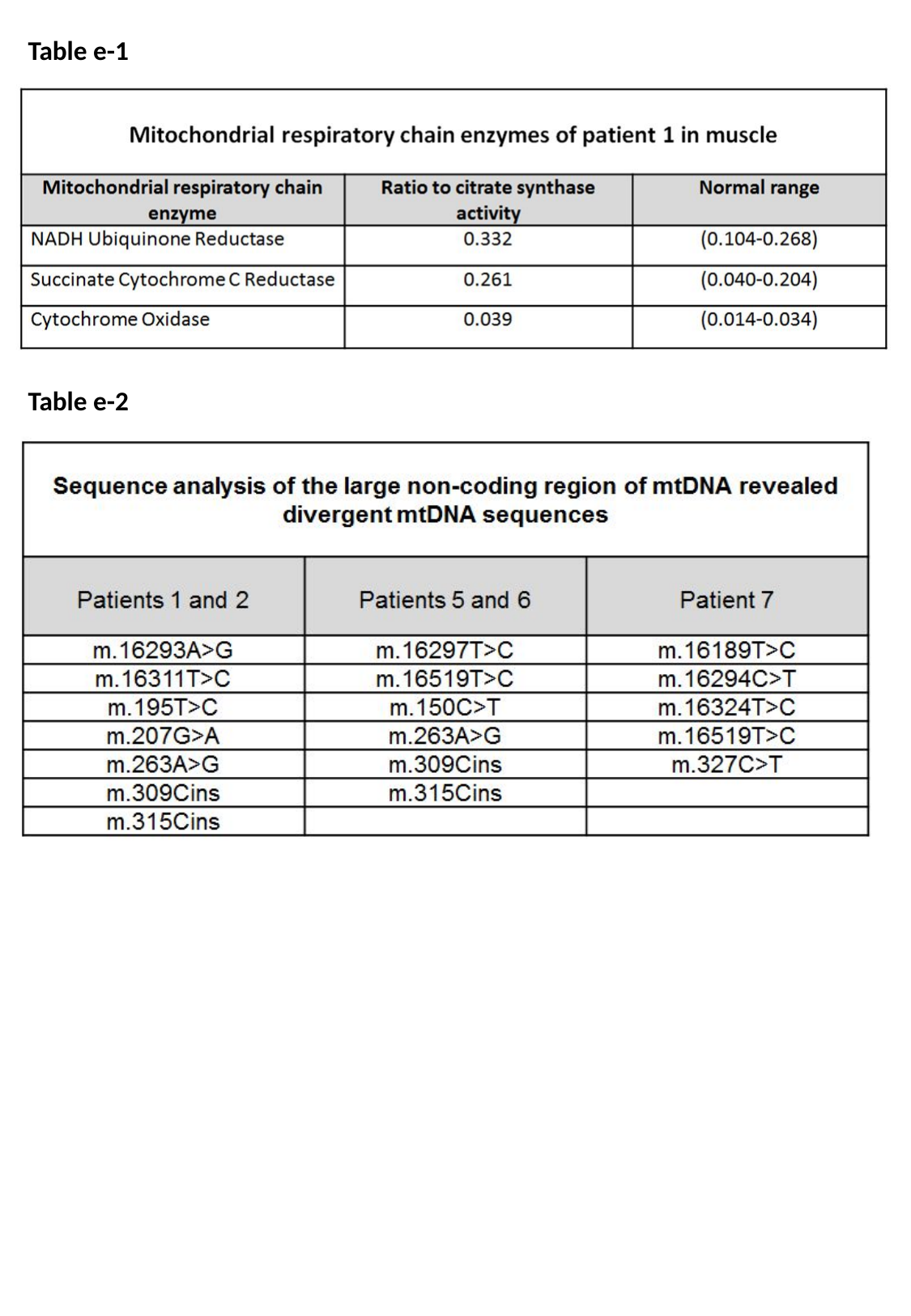

Table e-1
Table e-2
