# Supplementary material for: The m.13051G>A mitochondrial DNA mutation results in variable neurology and activated mitophagy
Source: Neurology. 2016 May 17;86(20):1921–3. doi: 10.1212/WNL.0000000000002688 (PMC4873683; doi:10.1212/WNL.0000000000002688)
Supplement: Data Supplement [file supp_WNL.0000000000002688_Supplementary_text.doc]

**Appendix e-1**

**Pathology**

**Patient 1**

She had auditory verbal agnosia and selective language regression due to bilateral sensorineural hearing loss at the age of 4 years old. MRI showed high signal in the basal ganglia and brainstem-suggesting necrosis. CSF lactate was raised. She presented to Eye Department at 5 years of age with very jerky smooth pursuit gaze horizontally and limited elevation of gaze with nystagmus and a right divergent squint with right dense amblyopia. Visual acuities were 1.5 for the right eye and 0.2 for the left eye. Normal pupil examination and normal electrophysiology exam with normal full field electroretinograms and normal visual evoked potentials. No deterioration was noted in visual acuity at her last examination at age 8 years.

**Patient 2**

Motor delay from 18 months onwards with subsequent learning difficulties. Intellectual development plateaued at the age of 10 years old. CSF lactate was raised. At the age of 11 there was no evidence of optic nerve disease with normal retinal and optic nerve examinations.

**Patient 3**

Acute onset of bilateral visual loss with a typical LHON phenotype at the age of 45 years old. At presentation the visual acuities were 0.62 for the right eye and 0.62 for the left eye. Visual fields showed significant loss of central fields extending to 20 degrees, with no recordable colour vision on Ishihara testing. Examination revealed optic nerve atrophy (Figure e-6).The patient’s visual acuities showed mild improvement after 2 years treatment with idebenone 300mg tds (0.44 for the right eye and 0.54 for the left eye).

**Patient 4**

Longstanding impaired vision, likely LHON.

**Patient 5**

Onset of generalized seizures at the age of 2 years old. Episodic abnormal breathing pattern, kyphosis and learning difficulties also developed in early childhood. Clinical examination at the age of 15 years old showed short stature, cognitive impairment, poor visual acuities with optic atrophy, pigmentary retinopathy, bilateral incomplete ophthalmoplegia, sensorineural deafness, cerebellar syndrome, brisk reflexes and ankle clonus. Serum and CSF lactate levels were both raised. The patient had persistent hyperkalaemia (6.6 – 7.4 mmol/L)

**Patient 6**

Acute onset of bilateral visual loss at the age of 15 years old. Final visual acuities were 0.125 in his right eye and 0.2 in his left eye with bilateral optic disc pallor. Eye movements were normal, but the patient had brisk lower limb reflexes. Serum lactate levels were normal.

**Relative 1**

The maternal grandmother was reviewed aged 72. She had been told in as a teenager that she had an optic nerve problem. She had noticed poor reading vision since then. Visual acuities were 0.9 right and 1.2 left and she was unable to see any colour plates on Ishihara testing. Goldmann visual fields were full.

**Relative 2**

Not available for examination: This man has ptosis.

**Relative 3**

Not available for examination: This man had learning difficulties in childhood, impaired hearing, and developed a movement disorder. Maximum walking distance aged 50 was 10 metres. Currently he has a “squint with jerky eye movements”.

**Patient 7**

A 43-year-old man presented with sudden onset, painless, central visual loss in his right eye. He was otherwise fit and well and there was no relevant family history. He was a non-smoker with no history of excessive alcohol intake. The patient’s visual acuities were documented at 0.05 in his right eye) and 1.0 in his left eye with a right dense central scotoma on Goldmann visual field perimetry. The right fundus appearance was classical of LHON showing optic disc hyperemia, peripapillary telangiectatic vessels and swelling of the nerve fiber layer. His neurological examination was normal and he was investigated extensively to document the cause of his right optic neuropathy. MRI neuroimaging and a lumbar puncture did not reveal any significant abnormalities. The primary LHON screen for the m.3460G>A, m.11778G>A and m.14484T>C came back as negative. Five months after the onset of visual loss in his right eye, the patient’s left eye became involved with rapid visual deterioration (Figure e-7A-B). The patient has not experienced any visual recovery and he has been registered legally blind with visual acuities stabilizing at hand movements in his right eye and count fingers in his left eye (Figure e-3C-D).

**Legend to Figure e-1**

Molecular assessment of the m.13051G>A mutation in muscle and blood from Patient 1 and blood from Patient 3. The analysis was performed using last-hot-cycle PCR-RFLP exploiting the loss of a HaeIII site due to the G>A nucleotide change, followed by gel electrophoresis. m.13051G>A MTND5 mutation analysis indicates a 99% mutant load in blood from both patients and a 100% mutant load in muscle from Patient 1.

**Legend to Figure e-2**

Blue Native Polyacrylamide Gel Electrophoresis Muscle respiratory chain complex activity stains of samples from Patient 1 show high protein levels of complexes when compared to control. The activity of Complex V and F1 subunits are comparable to control. Enzyme activities expressed as a ratio to citrate synthase activity are shown in Table e-1.

**Legend to Figure e-3**

Detection of reactive oxygen species (ROS) in fibroblasts derived from Patient 1 using Dihydrorhodamine 123 (DHR 123) showed a significantly increased ROS production when compared to three different control cell lines. Simultaneously, mitochondrial mass, measured by the cardiolipin specific dye 10 nonyl acridine orange (NAO), was also increased when compared to controls.

**Legend to Figure e-4**

Western blot analysis of fibroblast samples from Patient 1 showed increased levels of MnSOD (manganese superoxide dismutase), consistent with the increased ROS levels shown in Figure e-3.

**Legend to Figure e-5**

Additional analysis of patient derived fibroblasts with primary LHON mutations m.11778G>A (n=2) and m.3460G>A (n=1) and a compound heterozygote for mutations in the nuclear encoded ACAD9 gene (c.1150G>A, c.1168G>A) showed increased mitophagy at baseline compared to control. The increase in mitophagy seems to correlate with the severity of the phenotype (**** p<0.0001). Cells were grown in standard glucose media and measured using the In Cell1000 Analyzer.

**Legend to Figure e-6**

Examination of Patient 3 revealed optic nerve atrophy at the age of 45 years. At presentation visual acuities were 0.62 for the right eye and 0.62 for the left eye.

**Legend to Figure e-7**

Patient 7’s optic disc appearance in the acute and chronic stages. **Panel A and B**: Six months after first disease onset, there is residual retinal nerve fiber layer swelling and hyperaemia of the optic discs. Some peripapillary telangiectatic vessels can still be observed in the left eye, which was the second eye to be affected. Temporal optic disc pallor was already becoming apparent in the right eye. Visual acuities were count fingers in both eyes. **Panels** **C and D:** Fourteen months after first disease onset both optic discs had become atrophic with significant global thinning of the peripapillary retinal nerve fiber layer. Visual acuities were hand movements in the right eye and count fingers in the left eye.

LE: left eye; RE: right eye
